# Cytokines, chemokines and growth factors involved in keloids pathogenesis^☆^

**DOI:** 10.1016/j.abd.2024.01.010

**Published:** 2025-01-10

**Authors:** Mengguo Liu

**Affiliations:** Department of Dermatology, Huashan Hospital, Fudan University, Shanghai, China

**Keywords:** Keloid, Cytokines, Chemokines, Growth factors

## Abstract

Keloid is a common fibrotic disease, which is difficult to treat. It often causes itching and pain, which greatly disturbs patients in their work and daily life and causing difficulties in social interaction. Its pathogenesis is not clear, but may be related to several aspects: genetic susceptibility, environmental, immunological and endocrine factors, trauma and tension. The central point of its pathogenesis is the excessive proliferation of fibroblasts, with excessive synthesis and secretion of extracellular matrix such as collagen. However, the cause of fibroblast excessive proliferation and differentiation is not clear. Immune abnormalities may play an important role, with cytokines, chemokines, growth factors, and other important immune molecules acting on fibroblasts. This paper presents a detailed and comprehensive literature review on this subject.

## Introduction

Keloid is an excessive scarring that occurs after trauma or without obvious trauma. In terms of histopathology, keloid is mainly characterized by excessive deposition of extracellular matrix (ECM) mainly composed of collagen, which is beyond the scope of the original wound and invades the surrounding normal tissues. Keloid will progress slowly, often accompanied by itching or even pain, but also affect the appearance, often causing various physiological and psychological obstacles. At present, the exact mechanism of keloid is not completely clear, but the final result is excessive proliferation of fibroblasts in the dermis, increased synthesis, and reduced degradation of collagen and extracellular matrix. The imbalance between the two leads to excessive accumulation of collagen and fibrosis. In this complex process, there may be genetic susceptibility, tension factors, immune factors, endocrine factors, and other factors involved. Many previous studies have confirmed that immune factors play an important role in the pathogenesis of keloid, especially cytokines, chemokines and growth factors that may affect the functional changes of fibroblasts and participate in the pathogenesis of keloid by acting on fibroblasts. This presents makes a detailed and comprehensive review of this aspect, in order to find clues about the immune aspects of keloid. The authors searched for relevant literature on PubMed over the past 20 years, with keywords such as “keloids and cytokines, chemokines, growth factors”. The authors have systematically organized all the retrieved literature to form this review.

## Pro-inflammatory cytokines

### IL-1β

Interleukin-1 (IL-1), also known as lymphocyte stimulating factor, has two forms: IL-1α and IL-1β, which are mainly produced by monocyte and macrophages.^1^ Pro-inflammatory mediator IL-1 plays an important role in the early stage of scar formation. IL-1 is released following skin injury,^2^ which is known to activate fibroblasts^3^ and to stimulate the production of the prostaglandin E2 (PGE2) in human fibroblasts.^4^ PGE2 is a major eicosanoid product of fibroblasts, which has been shown to decrease fibroblast proliferation, and also has also been shown to reduce collagen levels by inhibiting its synthesis and promoting its degradation.^5^ IL-1β stimulates the production of PGE2 by both normal and keloid-derived fibroblasts. One study showed a reduced production of PGE2 by IL-1β stimulated keloid fibroblasts compared with those of control fibroblasts. In this study, although not statistically significant, IL-1β induced higher amounts of matrix metalloproteinase-1 (MMP-1) production by cultured skin fibroblasts derived from normal controls than those produced by keloid-derived skin fibroblasts. Since PGE2 may enhance the IL-1β-induced MMP-1 expression,^6^ a decrease in the production of PGE2 by keloid-derived fibroblasts may have implications in the decreased production of MMP-1 by keloid fibroblasts, which may contribute to the accumulation of extracellular matrix as seen in the keloid tissue. Results of a diminished production of PGE2 and MMP-1 by IL-1-stimulated keloid-derived fibroblasts may provide more information about factors contributing to keloid formation.

### IL-4

Research has shown that IL-4 is a major profibrotic cytokine that stimulates collagen synthesis by fibroblasts.^7^, ^8^, ^9^ In vitro stimulated by IL-4, human dermal fibroblasts synthesize a dose-dependent increase in pre-collagen mRNAs, resulting in elevated levels of types I and III collagen and fibronectin, which is neutralized by anti-IL-4 antibodies. IL-4 is strongly expressed during the early phases of normal wound healing in mouse skin and decreases after wound closure.^10^ Topical application of exogenous IL-4 on acute cutaneous wounds in mice induces a significant increase in the formation of fibrotic tissue, whereas the administration of IL-4 antisense oligonucleotides delays wound healing.^10^ Overexpression of IL-4 in transgenic mice results in fibroblast proliferation and increased focal deposition of collagen in the dermis.^11^ The expression of IL-4 was significantly different between patients with a family history of keloids and those with sporadic keloids. The high expression of IL-4 in the family history group may be of key importance in explaining why keloids run in families.^12^ RNA Sequencing Keloid Transcriptome showed significant up-regulation of IL-4R in the lesion of keloid patients compared to healthy skin. Furthermore, immunohistochemistry showed an increase of IL-4 r α^+^cell infiltration in keloid tissue.^13^

### IL-6

Many studies have shown that IL-6 plays an important role in the pathogenesis of fibroproliferative lesions that produce collagen. IL-6 produced by fibroblasts has been linked to the pathogenesis of fibrosis in abnormal wound-healing lesions such as keloid.^14^ Global gene expression analysis of keloid fibroblasts revealed the involvement of the interleukin-6 pathway.^15^ Ghazizadeh et al. report on their studies exploring the pathomechanisms of keloids with a focus on the IL-6 signaling pathway.^16^, ^17^ IL-6 and its receptors were significantly higher in keloid fibroblasts, with a concomitant increase in collagen biosynthesis. Anti-IL-6 antibodies or blocking the IL-6 receptors elicit reduced collagen synthesis, suggesting a role for IL-6 in the regulation of collagen gene expression. The mRNA and protein expressions of gp130 and several downstream targets in IL-6 signaling were upregulated in keloid fibroblasts. Other research shows that IL-6 serum levels were significantly higher in keloid patients.^18^ The higher serum levels of IL-6 were associated with the GG genotype, which was significantly higher in keloid patients and increased the risk for keloid development. A Japanese study showed the IL-6 polymorphism and susceptibility to keloid formation in a Japanese population.^19^ Their finding suggests that the IL-6 −572 G/C polymorphism is significantly associated with susceptibility to keloid formation and severity of keloid scarring, thus providing insight into strategies for the prediction and prevention of keloid formation. Similarly, another Chinese study also found that the IL-6 -572C > G polymorphism was closely associated with the incidence of keloid.^20^ These observations indicate that IL-6 signaling may play an integral role in keloid pathogenesis and provide clues for the development of IL-6 receptor-blocking strategies for therapy or prophylaxis of keloid scars.

### IL-8

IL-8 involved in keloid development and modulation of their expression may prove to be valuable in the long-term treatment of keloids. Keloid patients have higher peripheral blood endothelial progenitor cell counts and CD34^+^ cells with normal vasculogenic and angiogenic function that overexpress vascular endothelial growth factor (VEGF) and IL-8.^21^ Their data showed a robust increase in IL-8 gene expression in CD34^+^ cells, which was consistent with the increased VEGF gene activity in these cells. The effect might be reciprocal based on observations that IL-8 increases mRNA and synthesis of VEGF. These results support the role of vascular endothelial growth factor and interleukin-8 in the increased recruitment of endothelial progenitor cells in keloid patients. IL-8 functions as a neovascularization factor and is involved in angiogenesis.^22^ Another study found that the positive rate of IL-8 expression and its score in keloid were significantly higher than those in normal skin, the microvessel count in the tissues with IL-8 positive expression was higher than that in the tissues with IL-8 negative expression, and the expression score was positively correlated with the microvessel count, suggesting that fibroblasts and vascular endothelial cells of pathological keloid tissue synthesize and secrete IL-8, promoting the angiogenesis of keloid tissue, and leading to abnormal hyperplasia of keloid.^23^

### IL-13

IL-13 is a four-helix bundle cytokine located adjacent to IL-4 on chromosome 5q31. IL-13 shares several structural and functional characteristics with IL-4. IL-13 has been implicated in the pathogenesis of various diseases characterized by fibrosis. IL-13 modulates collagen homeostasis in human skin and keloid fibroblasts.^24^ Unlike IL-4 or transforming growth factor-β (TGF-β_1_), IL-13 specifically induced procollagen 3α1 gene expression. IL-13 induced marked up-regulation of total collagen and type I collagen generation from keloid fibroblasts. IL-13 was equipotent with IL-4 and TGF-β_1_ in this capacity. IL-13 might inhibit collagen degradation through inhibition of MMP-1 and MMP-3, promotion of Tissue Inhibitor of Metalloproteinase (TIMP)-1, enhancing collagen deposition. In abnormal scars, IL-4 and IL-13 induced periostin secretion, which in turn induces TGF-β_1_ secretion via the RhoA/ROCK pathway.^25^ TGF-β_1_ then induced further periostin production and secretion, and periostin and TGF-β_1_ cooperate to promote skin fibrosis in a ‘vicious cycle’. These findings suggested that the inhibition of IL-4/IL-13 and the RhoA/ROCK pathway might be potential therapeutic strategies to reduce skin fibrosis.

### IL-17

IL-17, a pro-inflammatory cytokine, is secreted by a distinct subtype of activated CD4^+^T-cells known as Th17.^26^, ^27^ Tumor-like stem cells derived from human keloids are governed by the inflammatory niche driven by the IL-17/IL-6 axis.^28^ A robust elevation of IL-6 and IL-17 expression in keloid is confirmed by cytokine array, western blot, and ELISA analyses. The altered biological functions are tightly regulated by the inflammatory niche mediated by an autocrine/ paracrine cytokine IL-17/IL-6 axis.^28^ Utilizing keloid-derived precursor cells transplanted subcutaneously in immunocompromised mice a human keloid-like tumor model that is driven by the in vivo inflammatory niche and allows testing of the anti-tumor therapeutic effect of antibodies targeting distinct niche components, specifically IL-6 and IL-17. IL-17 induced stromal cell-derived factor-1 and profibrotic factor in keloid-derived skin fibroblasts via the signal transducer and activator of transcription-3 (STAT3) pathway.^29^ Results showed that fibrotic reaction and expression of the proinflammatory cytokine IL-17 was most prominent in the growing margin (perilesional area) of keloid tissue and Th17 cells significantly infiltrated the perilesional area. In addition, IL-17 upregulated the expression of stromal cell-derived factor-1 (SDF-1), collagen, and α-smooth muscle actin (α-SMA) in keloid fibroblasts (KFs). The study demonstrated that a local increase in IL-17 in keloid tissues stimulates the production of SDF-1 in KFs causing further recruitment of Th17, which subsequently creates a positive feedback loop.^29^ These findings suggest that STAT3 inhibition can be used to treat keloid scars by reversing the vicious cycle between Th17 cells and KFs. IL-17 induces autophagy dysfunction to promote inflammatory cell death and fibrosis in keloid fibroblasts via the STAT3 and Hypoxia Inducible Factor-1α (HIF-1α) dependent signaling pathways.^30^ Defective autophagy caused by IL-17 was evaluated, and the relationship between defective autophagy and necroptosis was also examined. The expression of IL-17, HIF-1α, and STAT3 was significantly increased in keloid tissue, and autophagosome-to autophagolysosome conversion was defective in KF. IL-17 treatment significantly elevated the expression of STAT3 and HIF-1α in normal fibroblasts and caused defective autophagy, which was reversed by HIF-1α inhibitor. In addition, the defective autophagy was associated with the increased necroptosis and fibrosis. The polygenic map of keloid fibroblasts reveals fibrosis-associated gene alterations in inflammation and immune responses.^31^ This study reinforced the involvement of several immune cell subtypes and genes in fibrosis-related immune response pathways, including the IL-17.

### IL-18

IL-18 belongs to the proinflammatory cytokines, regulating inflammatory cell proliferation, and their secretion function, which suggests an important role in early inflammation.^32^ The expression of IL-18 was much higher in the keloid patients than that of the normal control group.^33^ IL-18 system plays an important role in keloid pathogenesis via epithelial-mesenchymal interactions.^34^ Not only IL-18, IL-18Rα and IL-18Rβ expression was also elevated in keloid tissue compared with normal skin tissue. Studies on the expression of IL-18 and its antagonist, IL-18 Binding Protein (IL-18BP), using a coculture model demonstrated severe IL-18⁄IL-18BP imbalance in keloid keratinocyte/keloid fibroblast cocultures with significant elevation of bioactive IL-18 whereas IL-18BP levels remained the same. Excessive secretion of mature IL-18 by keloid coculture and increased expression of IL-18R in keloid fibroblasts lead to enhanced secretion of collagen-ECM components and profibrotic cytokines such as IL-6 and IL-8. The addition of phosphatidylinositol 3-kinase (PI3K), mitogen activation protein kinase (MAPK), Specificity protein 1 (Sp1), and mammalian target of rapamycin (mTOR) inhibitors inhibited IL-18 secretion in keloid cocultures, suggesting their potential clinical use in the treatment of keloid.^34^

### IL-22

IL-22 is a cytokine mainly produced by T-cells and innate lymphoid cells. IL-22 primarily targets non-hematopoietic cells such as epithelial cells and fibroblasts. In the skin, IL-22 promotes the proliferation of keratinocytes and dermal fibroblasts.^35^ In the skin, IL-22 plays an important role in wound healing and is expressed after burn injury, being involved in tissue regeneration.^36^, ^37^, ^38^ IL-22 signaling is active in fibroblasts and directs expression of ECM genes and differentiation of myofibroblasts, and these physiological processes may become pathogenic if excessive matrix production destroys the normal tissue architecture and interferes with organ function, causing pathological scars.^39^ The number of relative copies of IL-22 mRNA was significantly higher in patients with keloids.^40^ Biopsies from normal and keloid scars were collected and the mRNA expression of several growth factors and cytokines were determined: TGF-β, fibroblast growth factor, IL-33, IL-22, arginase-1, arginase-2, inducible nitric oxide synthase, vasoactive intestinal peptide, and its receptor. Only IL-22, TGF-β, and arginase-1 exhibited significantly higher levels in keloid scars. The overexpression of IL-22, together with other molecule alterations, contributes to pathological scarring.

## Anti-inflammatory cytokines

### IL-10

One study showed that the expression of mRNA for IL-10 was lower significantly in keloid.^41^ Correlations between collagen type III and IL-10 were negative and significant. Another study described the therapeutic effect of interleukin-10 in keloid fibroblasts by suppression of TGF-β/Small mothers against the decapentaplegic (Smad) pathway.^42^ The study found that, compared with normal control, the proliferation of keloid fibroblasts was shown to be significantly suppressed on treatment with IL-10 in a time and dose-dependent manner. Expression of P-Smad 2/3 and Smad 4 were increasingly down-regulated, whereas Smad-7 was up-regulated with the increasing concentration of IL-10. By contrast, the variation of Smad 2/3 expressions was hardly influenced. Furthermore, collagen type I and collagen type II were found to be markedly decreased after treatment with IL-10. IL-10 inhibited the proliferation of keloid fibroblasts and collagen synthesis, and also IL-10 could negatively modulate the TGF-β/Smad signaling pathway, preventing keloid fibroblasts from proliferation and collagen production.^42^ These data suggest that IL-10 may have a potential role in the treatment of keloid.

### IL-24

IL-24 was originally identified as a tumor suppressor molecule, and then renamed IL-24 and classified as a cytokine, based on its chromosomal location in the IL-10 locus, its mRNA expression in leukocytes, and its secretory sequence elements.^43^ It was reported that the IL-24 gene might be involved in the formation of keloids.^44^ IL-24 mRNA in keloid fibroblasts was obviously lower than in normal skin. Adenovirus-mediated human interleukin 24 selectively suppresses proliferation and induces apoptosis in keloid fibroblasts.^45^ In this study, treatment with the replication-incompetent adenovirus vector carrying IL-24 gene selectively suppressed proliferation and induced apoptosis in keloid fibroblasts. Other studies showed that the lentivirus-mediated hlL-24 gene efficiently inhibits cell cycle progression, migration, and invasion activity of keloid fibroblasts.^46^ Thus, IL-24 has tremendous potential as a gene therapy for keloids and may lead to new gene therapeutics and provide a means of enhancing the therapeutic applications for keloid therapy.

### IL-37

IL-37 is a relatively new member of the IL-1 family, which is described as an anti-inflammatory mediator as occurs in various inflammatory autoimmune diseases.^47^ IL-37 has been issued as a novel anti-inflammatory cytokine that has extracellular and intracellular properties with eventual suppression of inflammation and innate immunity.^48^ IL-37 is involved in a negative feedback loop to control excess inflammation. In one study, there was a negative correlation between serum IL-37 level and the keloid severity.^49^ This negative correlation indicates that the lower level of serum IL-37, the more keloid severity. Thus, IL-37 may have a role in the pathogenesis of keloid owing to its ability to suppress the innate inflammatory immune responses.

## Chemokines^50^

Chemokines are a family of small proteins with 8‒10 KDa in size. Chemokines have been classified into four main subfamilies: C (XCL), CC (CCL), CXC (CXCL), and CX3C (CX3CL) chemokines. Chemokines are named by their ability to induce chemotaxis of nearby responsive cells. Chemokines play roles in many basic biological processes such as leukocyte trafficking and homing, organ development, angiogenesis, tumorigenesis and metastasis, inflammation, autoimmune response, and viral infection.

Recent data demonstrated that the recruitment of leukocyte subtypes was tightly regulated by chemokines during wound healing.^50^ In the hemostasis phase of cutaneous wound healing, CXCL4 appears to function by neutralization of heparin-like molecules on the endothelial surface of blood vessels, thereby inhibiting local antithrombin III activity and promoting coagulation.^50^ In the inflammation phase of cutaneous wound healing, in a mouse model of excisional skin, both wound-healing chemokine CX3CL1 and its receptor CX3CR1 were highly induced at wound sites. CX3CL1 colocalized with macrophages and endothelial cells, whereas CX3CR1 colocalized mainly with macrophages and fibroblasts.^51^ Loss of CX3CR1 function delayed wound closure in both CX3CR1 knockout and wild-type mice infused with anti-CX3CR1-neutralizing Ab.^51^ In the proliferation phase of cutaneous wound healing, CXCL11 was found to be a key ligand in the CXCR3 signaling system for wound repair, promoting re-epithelialization and modulating the maturation of the superficial dermis.^52^ CXCL11 simultaneously promotes re-epithelialization as a mediator of epidermal-dermal communication during wound repair.^53^ In the remodeling phase of cutaneous wound healing, important chemokines are CXCL11 produced by basal keratinocytes, and CXCL10 produced by neovascular endothelium, which interact with the chemokine receptor CXCR3. Stimulation of CXCR3 signaling converts fibroblasts from a migratory to a contractile state after an increase of mature dermal collagen fibers, increases keratinocyte migration by activation of m-calpain, and inhibits endothelial cell migration and proliferation.^54^ CCR2 signal was found in the keloid tissues.^55^ CXCL1 was present in myofibroblasts and lymphocytes in keloid tissues, which positively correlated with the degree of inflammatory infiltration in the lesions.^56^ Keloids also exhibited intensive immunoreactivity for the CXCR2 receptor in endothelial cells and inflammatory infiltrates with occasional staining of myofibroblasts.^57^ It can be inferred that in abnormal keloid scar formation, Chemokines derived from infiltrating cells in the dermis may further enhance cellular infiltrates and proinflammatory or fibrogenic cytokine release, leading to fibroblast activation.

## Growth factors

Keloid fibroblasts showed significantly greater growth response to epidermal growth factor (EGF) than normal fibroblasts. Procollagen type I carboxyterminal propeptide production was higher in keloid fibroblasts than in normal fibroblasts.^58^ VEGF has been implicated as a critical factor in regulating angiogenesis and inflammation under both physiological and pathological conditions. VEGF was upregulated in keloid fibroblasts compared with normal fibroblasts.^59^ VEGF may account for elevated levels of plasminogen activator inhibitor-1 via activating ERK1/2 in keloid fibroblasts.^60^ TGF-β is involved in inflammation, angiogenesis, proliferation of fibroblasts, collagen synthesis and extracellular matrix remodeling.^61^, ^62^, ^63^ TGF-β was expressed in dermal fibroblasts, inflammatory cells, and endothelial cells of keloids.^64^, ^65^ TGF-β induces polypyrimidine tract-binding protein to alter fibroblasts proliferation and fibronectin deposition in keloid.^66^ TGF-β, initiated epithelial-mesenchymal transition (EMT) in keloid epithelial cells by inducing the up-regulation of snail2, and TGF-β/Smad 3 signaling pathway was involved in EMT. EMT could change the phenotype of epithelial stem cells in keloid. Activating transcription factor 3 regulates cell growth, apoptosis, invasion and collagen synthesis in keloid fibroblast TGF-β/Smad signaling pathway.^67^ On the contrary, basic fibroblast growth factor (bFGF) reduced keloid and promoted wound healing by inhibiting TGFβ1/Smad-dependent pathway.^68^ bFGF exhibited significant amelioration of the collagen tissue. bFGF regulated ECM synthesis and degradation via interference in the collagen distribution. bFGF may be a potential new therapeutic tool for the treatment of hypertrophic and keloid scars. Different types of growth factors play different roles in keloid formation, and their specific roles need to be further explored.

## Conclusion

**Figure 1:**
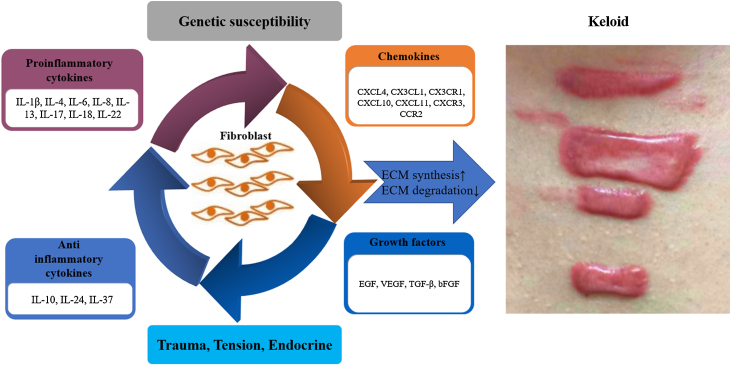
**Schematic representation of key factors in keloid pathogenesis.** The imbalance of proinflammatory and anti-inflammatory cytokines exists in all stages of wound healing, acting on skin fibroblasts, involving in skin tissue remodeling, and promoting the formation of keloids in severe conditions. Chemokines and growth factors also contribute to inflammatory processes, stimulating the chemotaxis of inflammatory cells that then further secrete proinflammatory cytokines, and stimulate fibroblasts, thus creating a vicious circle that poses a major challenge in treating and slowing the progression of keloid. bFGF, basic Fibroblast Growth Factor; ECM, Extracellular Matrix; EGF, Epidermal Growth Factor; IL, Interleukin; TGF-β, Transforming Growth Factor-β; VEGF, Vascular Endothelial Growth Factor.

**Figure 2:**
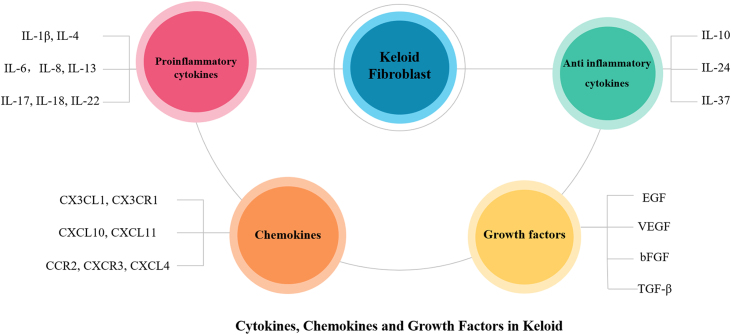
**Cytokines, Chemokines and Growth Factors in Keloid**. There are many different kinds of cytokines, chemokines and growth factors involved in the pathogenesis of keloid.

This article reviews in detail the possible roles of proinflammatory cytokines (IL-1β, IL-4, IL-6, IL-8, IL-13, IL-17, IL-18, IL-22), anti-inflammatory cytokines (IL-10, IL-24, IL-37), chemokines (CXCL4, CX3CL1, CX3CR1, CXCL10, CXCL11, CXCR3, CCR2) and growth factors (EGF, VEGF, TGF-β, bFGF) in the pathogenesis of keloid (Figure 1, Figure 2). In the future, the authors can conduct more in-depth and thorough research on them, select more specific factors related to the pathogenesis of keloid, which may be used as new therapeutic targets in the future to provide new ideas for the treatment of keloid.

## Financial support

This work was supported by grants from the National Natural Science Foundation of China (81602747).

## Author’ contributions

Mengguo Liu: Conceived the review idea, wrote, proofread and edited the manuscript.

## Conflicts of interest

None declared.
